# The genome sequence of the Olive Pearl, *Udea olivalis* (Denis & Schiffermüller, 1775)

**DOI:** 10.12688/wellcomeopenres.19373.1

**Published:** 2023-06-12

**Authors:** Douglas Boyes, Peter W.H. Holland, University of Oxford and Wytham Woods Genome Acquisition Lab, Darwin Tree of Life Barcoding collective, Wellcome Sanger Institute Tree of Life programme, Wellcome Sanger Institute Scientific Operations: DNA Pipelines collective, Tree of Life Core Informatics collective, Darwin Tree of Life Consortium

**Affiliations:** 1 UK Centre for Ecology & Hydrology, Wallingford, England, UK; 2 University of Oxford, Oxford, England, UK

**Keywords:** Udea olivalis, the Olive Pearl, genome sequence, chromosomal, Lepidoptera

## Abstract

We present a genome assembly from an individual male *Udea olivalis* (the Olive Pearl; Arthropoda; Insecta; Lepidoptera; Crambidae). The genome sequence is 624.4 megabases in span. Most of the assembly is scaffolded into 31 chromosomal pseudomolecules, including the Z sex chromosome. The mitochondrial genome has also been assembled and is 15.3 kilobases in length.

## Species taxonomy

Eukaryota; Metazoa; Ecdysozoa; Arthropoda; Hexapoda; Insecta; Pterygota; Neoptera; Endopterygota; Lepidoptera; Glossata; Ditrysia; Pyraloidea; Crambidae; Spilomelinae; *Udea*; *Udea olivalis* (Denis & Schiffermüller, 1775) (NCBI:txid1002971).

## Background

The superfamily Pyraloidea includes over 15,000 species of moths adapted to a wide diversity of habitats. Molecular phylogenetic analysis divides the superfamily into two sister clades, each usually given family status: the Pyralidae and Crambidae ( Regier *et al.*, 2012). *Udea olivalis*, sometimes given the common name the Olive Pearl, is a widespread member of the latter clade. Similar to several related ‘crambids’, *U. olivalis* holds its wings at rest in a flat delta shape, clearly showing the grey-brown ground colour of the forewings marked with cream spots, including a diagnostic central trapezoid shape ( Asher *et al.*, 2013).

*U. olivalis* is distributed patchily across northern and central Europe and can be locally common in southern England, Wales, Northern Ireland, lowland regions of Scotland and eastern regions of Ireland ( GBIF Secretariat, 2022; NBN Atlas Partnership, 2021; National Biodiversity Data Centre, 2023). The moth is found in woodlands, hedgerows and suburban gardens and in some regions is more frequent in woodlands on calcareous soils ( Davey, 2019). The larvae feed on a wide range of herbaceous plants, including woundworts *Stachys* sp., nettle *Urtica dioica*, ground ivy *Nepeta hederacea*, dog’s mercury *Mercurialis perennis*, dock *Rumex* sp. and hop *Humulus lupulus* ( Beirne, 1952). In Britain and Ireland, the species is univoltine, with the adults on the wing primarily in June and July ( Asher *et al.*, 2013; NBN Atlas Partnership, 2021; National Biodiversity Data Centre, 2023).

A genome sequence for *U. olivalis* will facilitate studies investigating adaptations to polyphagy and contribute to the growing set of genomic resources for understanding the evolutionary diversification of Lepidoptera.

## Genome sequence report

**Figure 1.**
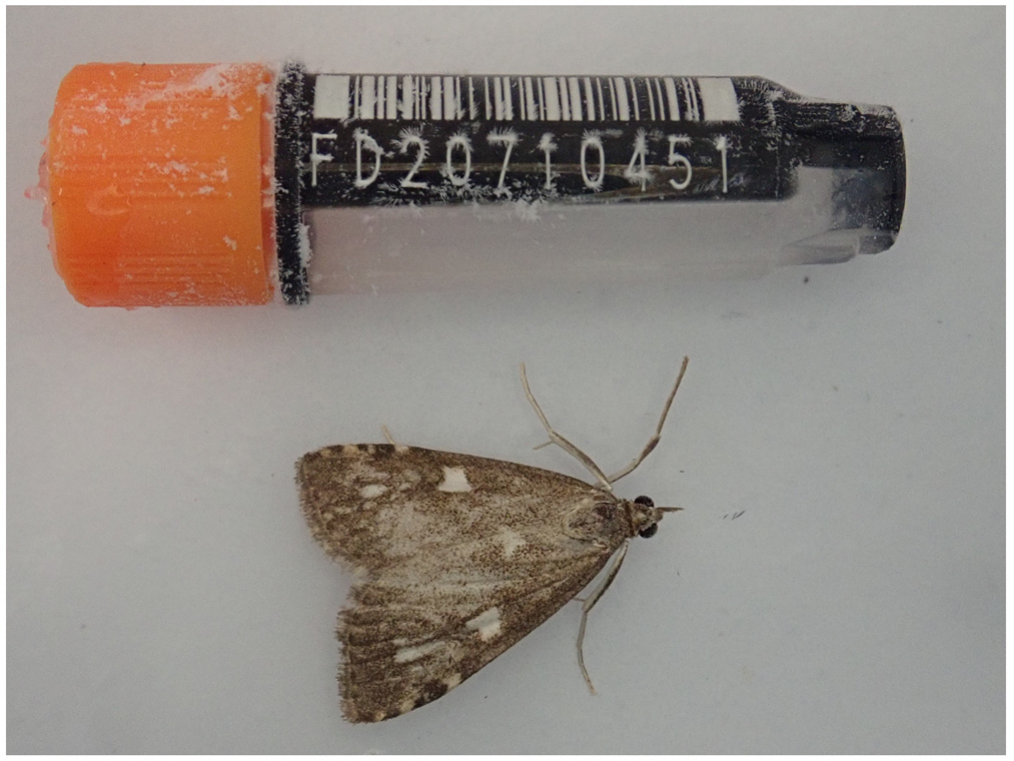
Photograph of the *Udea olivalis* (ilUdeOliv2) specimen used for genome sequencing.

The genome was sequenced from one male *Udea olivalis* ( Figure 1) collected from Wytham Woods, Oxfordshire (biological vice-county: Berkshire), UK (latitude 51.77, longitude –1.34). A total of 36-fold coverage in Pacific Biosciences single-molecule HiFi long reads was generated. Primary assembly contigs were scaffolded with chromosome conformation Hi-C data. Manual assembly curation corrected 19 missing joins or mis-joins and removed one haplotypic duplication, reducing the scaffold number by 16.07%.

**Figure 2.**
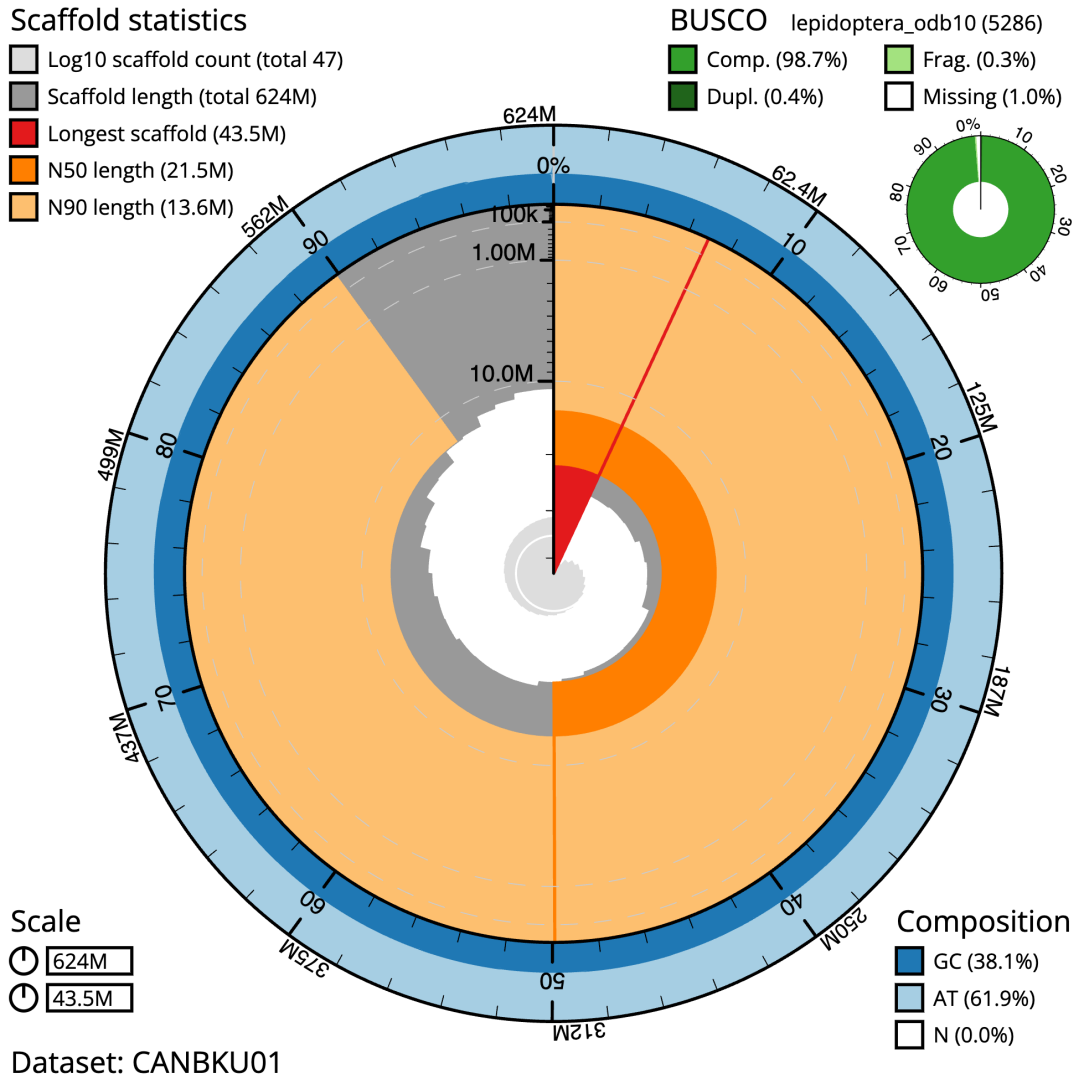
Genome assembly of *Udea olivalis*, ilUdeOliv2.1: metrics. The BlobToolKit Snailplot shows N50 metrics and BUSCO gene completeness. The main plot is divided into 1,000 size-ordered bins around the circumference with each bin representing 0.1% of the 624,353,836 bp assembly. The distribution of scaffold lengths is shown in dark grey with the plot radius scaled to the longest scaffold present in the assembly (43,521,136 bp, shown in red). Orange and pale-orange arcs show the N50 and N90 scaffold lengths (21,486,546 and 13,554,949 bp), respectively. The pale grey spiral shows the cumulative scaffold count on a log scale with white scale lines showing successive orders of magnitude. The blue and pale-blue area around the outside of the plot shows the distribution of GC, AT and N percentages in the same bins as the inner plot. A summary of complete, fragmented, duplicated and missing BUSCO genes in the lepidoptera_odb10 set is shown in the top right. An interactive version of this figure is available at https://blobtoolkit.genomehubs.org/view/ilUdeOliv2.1/dataset/CANBKU01/snail.

The final assembly has a total length of 624.4 Mb in 47 sequence scaffolds with a scaffold N50 of 21.5 Mb ( Table 1). Most (99.87%) of the assembly sequence was assigned to 31 chromosomal-level scaffolds, representing 30 autosomes, and the Z sex chromosome. Chromosome-scale scaffolds confirmed by the Hi-C data are named in order of size ( Figure 2– Figure 5; Table 2). While not fully phased, the assembly deposited is of one haplotype. Contigs corresponding to the second haplotype have also been deposited. The mitochondrial genome was also assembled and can be found as a contig within the multifasta file of the genome submission.

**Table 1.** Genome data for *Udea olivalis*, ilUdeOliv2.1.

Project accession data		
Assembly identifier	ilUdeOliv2.1	
Species	*Udea olivalis*	
Specimen	ilUdeOliv2	
NCBI taxonomy ID	1002971	
BioProject	PRJEB56567	
BioSample ID	SAMEA10979198	
Isolate information	ilUdeOliv2, male (genome sequencing, Hi-C scaffolding)	
Assembly metrics *		*Benchmark*
Consensus quality (QV)	65.7	*≥ 50*
*k*-mer completeness	100%	*≥ 95%*
BUSCO **	C:98.7%[S:98.4%,D:0.4%], F:0.3%,M:1.0%,n:5,286	*C ≥ 95%*
Percentage of assembly mapped to chromosomes	99.87%	*≥ 95%*
Sex chromosomes	Z chromosome	*localised homologous pairs*
Organelles	Mitochondrial genome assembled	*complete single alleles*
Raw data accessions		
PacificBiosciences SEQUEL II	ERR10368986	
Hi-C Illumina	ERR10323161	
Genome assembly		
Assembly accession	GCA_947369235.1	
*Accession of alternate haplotype*	GCA_947369245.1	
Span (Mb)	624.4	
Number of contigs	140	
Contig N50 length (Mb)	8.2	
Number of scaffolds	47	
Scaffold N50 length (Mb)	21.5	
Longest scaffold (Mb)	43.5	

* Assembly metric benchmarks are adapted from column VGP-2020 of “Table 1: Proposed standards and metrics for defining genome assembly quality” from ( Rhie *et al.*, 2021). ** BUSCO scores based on the lepidoptera_odb10 BUSCO set using v5.3.2. C = complete [S = single copy, D = duplicated], F = fragmented, M = missing, n = number of orthologues in comparison. A full set of BUSCO scores is available at https://blobtoolkit.genomehubs.org/view/ilUdeOliv2.1/dataset/CANBKU01/busco.

**Figure 3.**
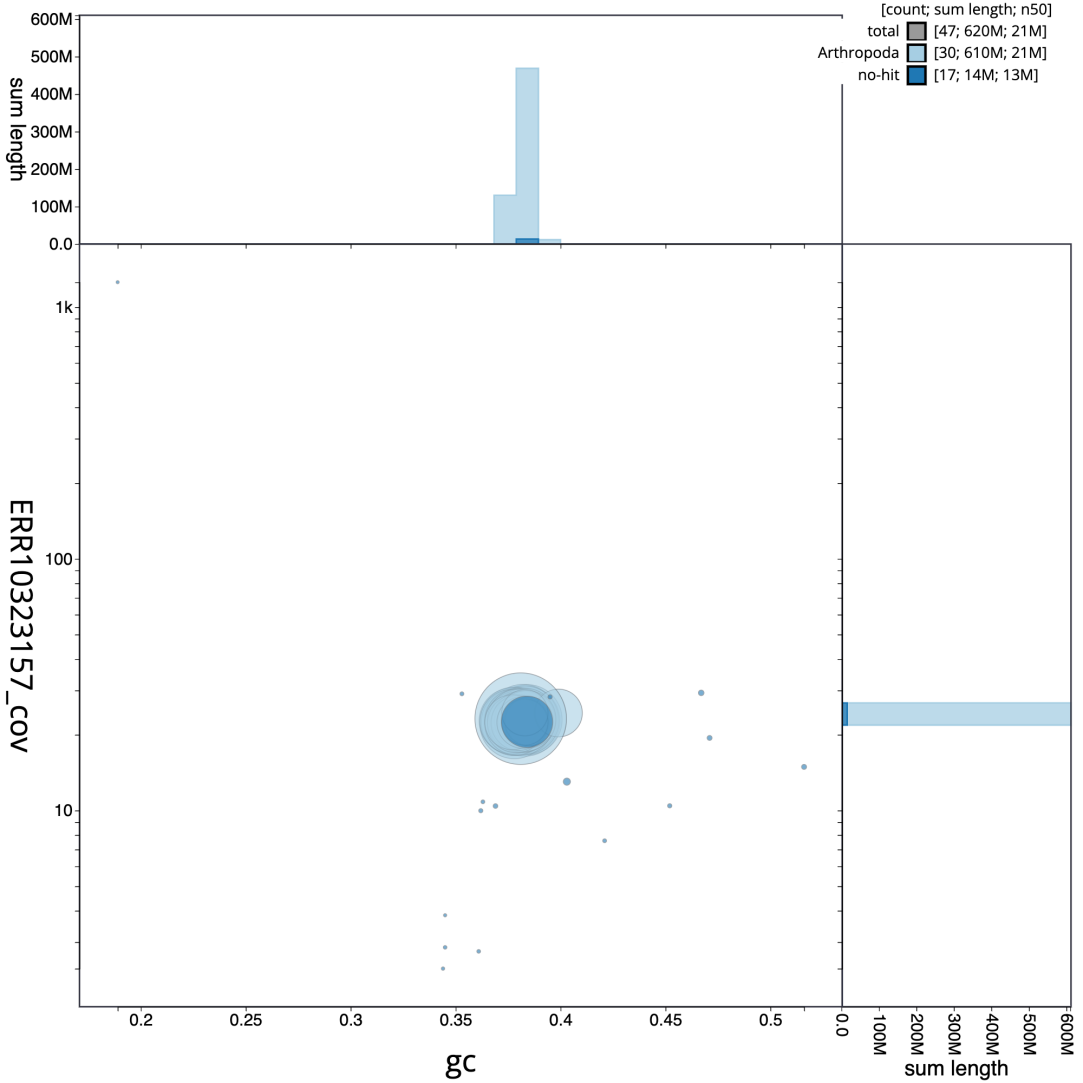
Genome assembly of *Udea olivalis*, ilUdeOliv2.1: GC coverage. BlobToolKit GC-coverage plot. Scaffolds are coloured by phylum. Circles are sized in proportion to scaffold length. Histograms show the distribution of scaffold length sum along each axis. An interactive version of this figure is available at https://blobtoolkit.genomehubs.org/view/ilUdeOliv2.1/dataset/CANBKU01/blob.

**Figure 4.**
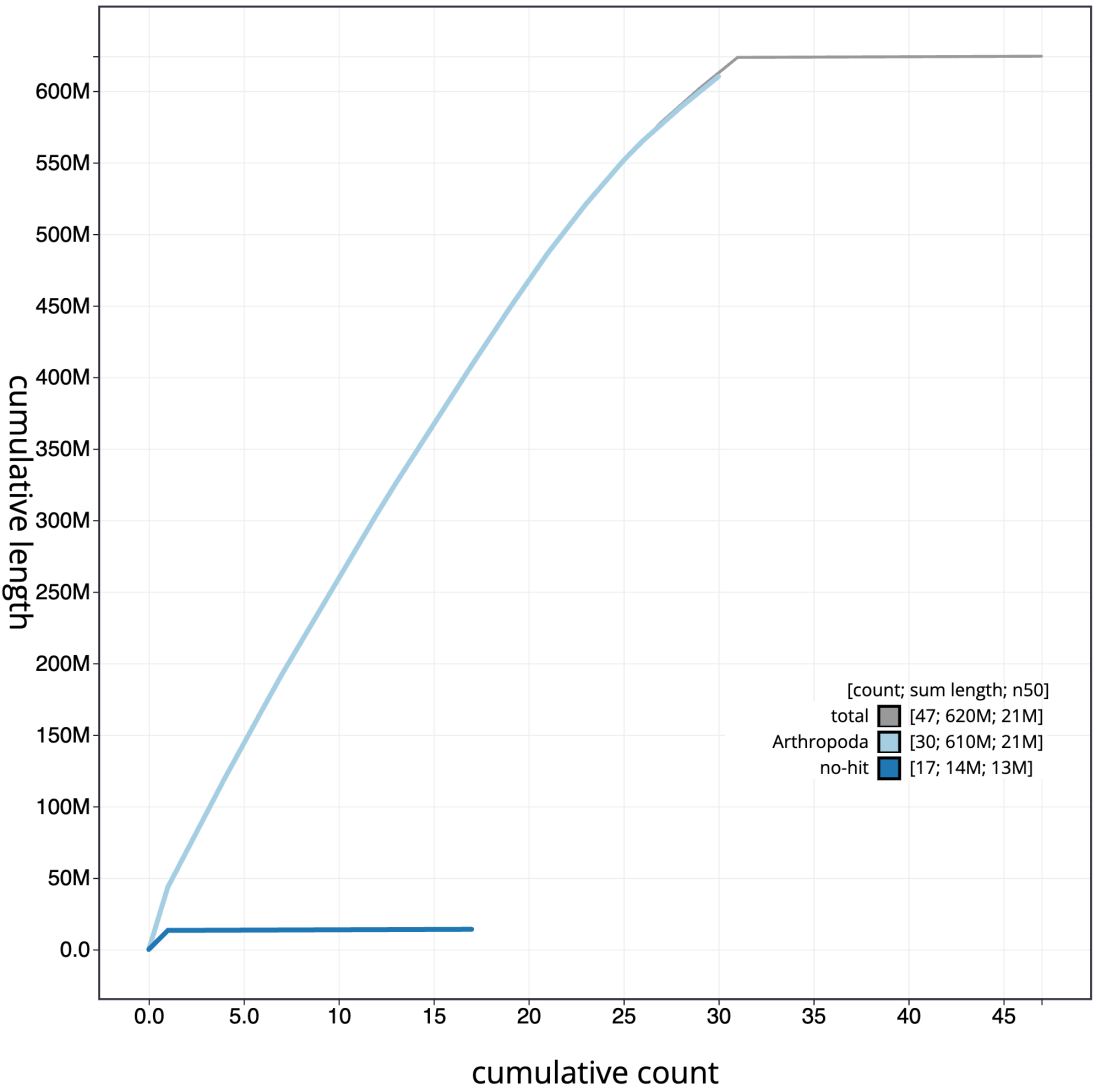
Genome assembly of *Udea olivalis*, ilUdeOliv2.1: cumulative sequence. BlobToolKit cumulative sequence plot. The grey line shows cumulative length for all scaffolds. Coloured lines show cumulative lengths of scaffolds assigned to each phylum using the buscogenes taxrule. An interactive version of this figure is available at https://blobtoolkit.genomehubs.org/view/ilUdeOliv2.1/dataset/CANBKU01/cumulative.

**Figure 5.**
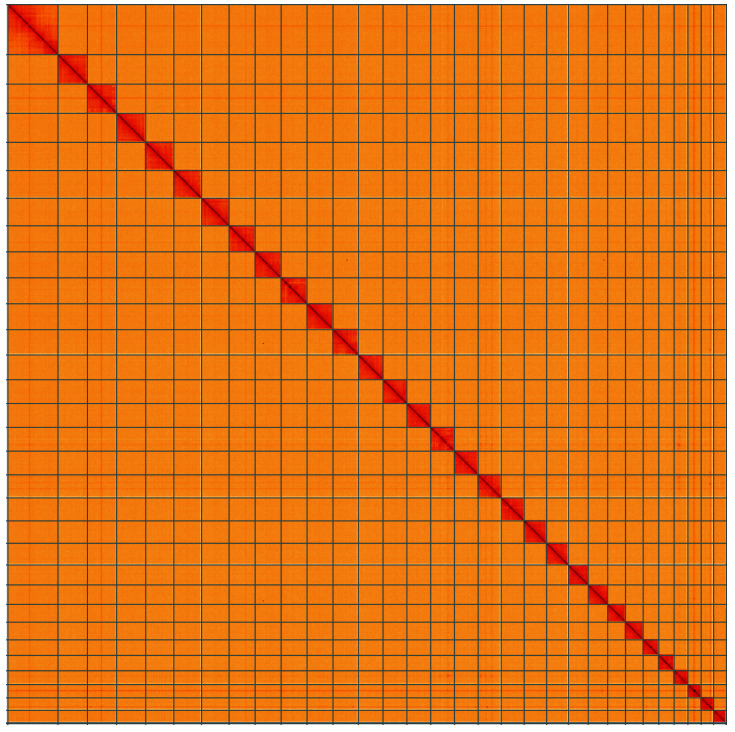
Genome assembly of *Udea olivalis*, ilUdeOliv2.1: Hi-C contact map. Hi-C contact map of the ilUdeOliv2.1 assembly, visualised using HiGlass. Chromosomes are shown in order of size from left to right and top to bottom. An interactive version of this figure may be viewed at https://genome-note-higlass.tol.sanger.ac.uk/l/?d=Aa99HtjxT8uJIgZIkqHk5A.

**Table 2.** Chromosomal pseudomolecules in the genome assembly of *Udea olivalis*, ilUdeOliv2.

INSDC accession	Chromosome	Size (Mb)	GC%
OX376341.1	1	25.56	38.3
OX376342.1	2	25.35	38.4
OX376343.1	3	25.25	38.2
OX376344.1	4	24.42	37.8
OX376345.1	5	24.06	38.3
OX376346.1	6	23.93	37.8
OX376347.1	7	22.6	37.9
OX376348.1	8	22.58	38
OX376349.1	9	22.5	37.7
OX376350.1	10	22.5	37.8
OX376351.1	11	21.97	38.1
OX376352.1	12	21.49	37.7
OX376353.1	13	20.71	38.1
OX376354.1	14	20.68	37.9
OX376355.1	15	20.63	37.9
OX376356.1	16	20.56	38.1
OX376357.1	17	20.04	38.3
OX376358.1	18	19.95	38
OX376359.1	19	19.45	38.2
OX376360.1	20	18.85	38.3
OX376361.1	21	17.25	37.9
OX376362.1	22	16.93	38.4
OX376363.1	23	15.47	38
OX376364.1	24	15.31	37.7
OX376365.1	25	13.55	38.2
OX376366.1	26	13.32	38.4
OX376367.1	27	11.99	38.5
OX376368.1	28	11.52	39.9
OX376369.1	29	10.82	38.3
OX376370.1	30	10.8	38.3
OX376340.1	Z	43.52	38.1
OX376371.1	MT	0.02	19.2

The estimated Quality Value (QV) of the final assembly is 65.7 with *k*-mer completeness of 100%, and the assembly has a BUSCO v5.3.2 completeness of 98.7% (single = 98.4%, duplicated = 0.4%), using the lepidoptera_odb10 reference set ( *n* = 5,286).

Metadata for specimens, spectral estimates, sequencing runs, contaminants and pre-curation assembly statistics can be found at https://links.tol.sanger.ac.uk/species/1002971.

## Methods

### Sample acquisition and nucleic acid extraction

A male *Udea olivalis* (ilUdeOliv2) was collected from Wytham Woods, Oxfordshire (biological vice-county: Berkshire), UK (latitude 51.77, longitude –1.34) on 16 June 2021. The specimen was taken from the orchard by Douglas Boyes (University of Oxford) using a net. The specimen was identified by the collector and snap-frozen on dry ice.

The ilUdeOliv2 sample was prepared at the Tree of Life laboratory, Wellcome Sanger Institute (WSI). the sample was weighed and dissected on dry ice with tissue set aside for Hi-C sequencing. Whole organism tissue was disrupted using a Nippi Powermasher fitted with a BioMasher pestle. DNA was extracted at the WSI Scientific Operations core using the Qiagen MagAttract HMW DNA kit, according to the manufacturer’s instructions.

### Sequencing

Pacific Biosciences HiFi circular consensus DNA sequencing libraries were constructed according to the manufacturers’ instructions. DNA sequencing was performed by the Scientific Operations core at the WSI on the Pacific Biosciences SEQUEL II (HiFi) instrument. Hi-C data were also generated from tissue of ilUdeOliv2 that was set aside for the purpose using the Arima2 kit and sequenced on the Illumina NovaSeq 6000 instrument.

### Genome assembly, curation and evaluation

Assembly was carried out with Hifiasm ( Cheng *et al.*, 2021) and haplotypic duplication was identified and removed with purge_dups ( Guan *et al.*, 2020). The assembly was then scaffolded with Hi-C data ( Rao *et al.*, 2014) using YaHS ( Zhou *et al.*, 2023). The assembly was checked for contamination and corrected using the gEVAL system ( Chow *et al.*, 2016) as described previously ( Howe *et al.*, 2021). Manual curation was performed using gEVAL, HiGlass ( Kerpedjiev *et al.*, 2018) and Pretext ( Harry, 2022). The mitochondrial genome was assembled using MitoHiFi ( Uliano-Silva *et al.*, 2022), which runs MitoFinder ( Allio *et al.*, 2020) or MITOS ( Bernt *et al.*, 2013) and uses these annotations to select the final mitochondrial contig and to ensure the general quality of the sequence. To evaluate the assembly, MerquryFK was used to estimate consensus quality (QV) scores and *k*-mer completeness ( Rhie *et al.*, 2020). The genome was analysed within the BlobToolKit environment ( Challis *et al.*, 2020) and BUSCO scores ( Manni *et al.*, 2021; Simão *et al.*, 2015) were calculated. Table 3 contains a list of software tool versions and sources.

**Table 3.** Software tools: versions and sources.

Software tool	Version	Source
BlobToolKit	4.0.7	https://github.com/blobtoolkit/blobtoolkit
BUSCO	5.3.2	https://gitlab.com/ezlab/busco
gEVAL	N/A	https://geval.org.uk/
Hifiasm	0.16.1-r375	https://github.com/chhylp123/hifiasm
HiGlass	1.11.6	https://github.com/higlass/higlass
Merqury	MerquryFK	https://github.com/thegenemyers/MERQURY.FK
MitoHiFi	2	https://github.com/marcelauliano/MitoHiFi
PretextView	0.2	https://github.com/wtsi-hpag/PretextView
purge_dups	1.2.3	https://github.com/dfguan/purge_dups
YaHS	yahs-1.1.91eebc2	https://github.com/c-zhou/yahs

### Ethics and compliance issues

The materials that have contributed to this genome note have been supplied by a Darwin Tree of Life Partner. The submission of materials by a Darwin Tree of Life Partner is subject to the Darwin Tree of Life Project Sampling Code of Practice. By agreeing with and signing up to the Sampling Code of Practice, the Darwin Tree of Life Partner agrees they will meet the legal and ethical requirements and standards set out within this document in respect of all samples acquired for, and supplied to, the Darwin Tree of Life Project. All efforts are undertaken to minimise the suffering of animals used for sequencing. Each transfer of samples is further undertaken according to a Research Collaboration Agreement or Material Transfer Agreement entered into by the Darwin Tree of Life Partner, Genome Research Limited (operating as the Wellcome Sanger Institute), and in some circumstances other Darwin Tree of Life collaborators.

## Data Availability

European Nucleotide Archive: *Udea olivalis* (olive pearl). Accession number PRJEB56567; https://identifiers.org/ena.embl/PRJEB56567. ( Wellcome Sanger Institute, 2022) The genome sequence is released openly for reuse. The *Udea olivalis* genome sequencing initiative is part of the Darwin Tree of Life (DToL) project. All raw sequence data and the assembly have been deposited in INSDC databases. The genome will be annotated using available RNA-Seq data and presented through the Ensembl pipeline at the European Bioinformatics Institute. Raw data and assembly accession identifiers are reported in Table 1.

## Author information

Members of the University of Oxford and Wytham Woods Genome Acquisition Lab are listed here: https://doi.org/10.5281/zenodo.4789928.

Members of the Darwin Tree of Life Barcoding collective are listed here: https://doi.org/10.5281/zenodo.4893703.

Members of the Wellcome Sanger Institute Tree of Life programme are listed here: https://doi.org/10.5281/zenodo.4783585.

Members of Wellcome Sanger Institute Scientific Operations: DNA Pipelines collective are listed here: https://doi.org/10.5281/zenodo.4790455.

Members of the Tree of Life Core Informatics collective are listed here: https://doi.org/10.5281/zenodo.5013541.

Members of the Darwin Tree of Life Consortium are listed here: https://doi.org/10.5281/zenodo.4783558.
