# Supplementary material for: The genome sequence of the Olive Pearl, Udea olivalis (Denis & Schiffermüller, 1775)
Source: Wellcome Open Res. 2023 Jun 12;8:238. [Version 1] doi: 10.12688/wellcomeopenres.19373.1 (PMC11487470; doi:10.12688/wellcomeopenres.19373.1)
Supplement: Peer review history

# Reviewer report: Reviewer response for version 1

**DOI:** 10.21956/wellcomeopenres.21460.r102482

**Published:** 2024-10-17

**Authors:** Michal Rindoš

**Affiliations:** 1 University of South Bohemia, Ceske Budejovice, Czech Republic

To be honest, that Abstract can be improved, definitively this should not included - ; Arthropoda; Insecta; Lepidoptera; Crambidae. 

 Nepeta herecacea is synonym of Glechoma hederacea, which is a valid name 

 Otherwise I am fine with the facts stated and also with the quality of the genome.

 They could of course write something more about the genus Udea in general.

Are sufficient details of methods and materials provided to allow replication by others?

Yes

Is the rationale for creating the dataset(s) clearly described?

Yes

Are the datasets clearly presented in a useable and accessible format?

Yes

Are the protocols appropriate and is the work technically sound?

Yes

Reviewer Expertise:

Lepidoptera Biodiversity and Sytematics

I confirm that I have read this submission and believe that I have an appropriate level of expertise to confirm that it is of an acceptable scientific standard, however I have significant reservations, as outlined above.

# Reviewer report: Reviewer response for version 1

**DOI:** 10.21956/wellcomeopenres.21460.r102498

**Published:** 2024-10-15

**Authors:** Melody Clark

**Affiliations:** 1 Natural Environment Research Council, Cambridge, UK

This paper describes the high quality genome sequence of an individual male *Udea olivalis. *This common UK moth was sequenced as part of the Darwin Tree of Life Project and is another small piece of the jig-saw that is the Wytham Woods ecosystem. Long-term research has been conducted in Wytham Woods since the 1940s and it is one of the most researched woodlands in the world. Sequencing all the biota in this wood presents a phenomenal opportunity to link genomics and evolution with ecology and long-term monitoring. This new moth genome will aid in elucidating the evolution of the Lepidoptera, more particularly should provide insight into the adaptation of a species to exploit on a wide variety of food stuffs (as is the case with this species). The genome is of high quality and has been produced and analysed using the Darwin Tree of Life pipeline.

Are sufficient details of methods and materials provided to allow replication by others?

Yes

Is the rationale for creating the dataset(s) clearly described?

Yes

Are the datasets clearly presented in a useable and accessible format?

Yes

Are the protocols appropriate and is the work technically sound?

Yes

Reviewer Expertise:

Transcriptomics and genomics of non-model species

I confirm that I have read this submission and believe that I have an appropriate level of expertise to confirm that it is of an acceptable scientific standard.

# Reviewer report: Reviewer response for version 1

**DOI:** 10.21956/wellcomeopenres.21460.r79116

**Published:** 2024-09-24

**Authors:** Tobias Baril

**Affiliations:** 1 University of Exeter, Exeter, England, UK

The authors present a near chromosome-level genome assembly for the Olive Pearl, Udea olivalis. A mitochondrial contig and reads corresponding to the second haplotype are also deposited. The genome is sequenced from a single male, which means there is no W chromosome sequence provided.

 The report is straightforward and well written with clear description of the results and methods used including bioinformatics software with version information where appropriate.

 Minor comments

 - Figure 3 contains a reference to dataset ERR10323157, which is not explained in the figure legend and is also not a dataset described in Table 1, where the only similar IDs are ERR10323161 and ERR10368986. Can the authors confirm the source of this ID and provide information on the dataset that it references?

Are sufficient details of methods and materials provided to allow replication by others?

Yes

Is the rationale for creating the dataset(s) clearly described?

Yes

Are the datasets clearly presented in a useable and accessible format?

Yes

Are the protocols appropriate and is the work technically sound?

Yes

Reviewer Expertise:

Genomics, Bioinformatics, Genome Assembly, Transposable elements, Population Genetics

I confirm that I have read this submission and believe that I have an appropriate level of expertise to confirm that it is of an acceptable scientific standard.
